# Genome-Wide Identification of the GRAS Transcription Factor Family in *Coptis chinensis* Reveals Tissue-Specific Co-Expression with bZIP Proteins Under Light Stress

**DOI:** 10.3390/ijms27104617

**Published:** 2026-05-21

**Authors:** Wuke Wei, Jun Tan, Lianan Guo, Yili Zhang, Yu Wang, Yuan Pan

**Affiliations:** 1 Chongqing Academy of Chinese Materia Medica, Chongqing 400065, China; wk0213@gzucm.edu.cn (W.W.); qaz5856-plm1186@163.com (J.T.); guolianan_2026@163.com (L.G.); 2 Ministry of Education Key Laboratory of Chinese Medicinal Resource from Lingnan, School of Pharmaceutical Sciences, Guangzhou University of Chinese Medicine, Guangzhou 510006, China; 3 School of Chinese Materia Medica, Chongqing University of Chinese Medicine, Chongqing 401121, China; 4 Chongqing Institute for Food and Drug Control, Chongqing 402760, China; zyl315607796@163.com

**Keywords:** *Coptis chinensis*, GRAS transcription factor, bZIP transcription factor, light stress, tissue-specific expression, co-regulation, genome-wide identification

## Abstract

GRAS transcription factors are essential for plant growth and stress adaptation, yet they remain uncharacterized in the medicinal herb *Coptis chinensis*. To address this gap, we performed a genome-wide identification of the GRAS family and investigated its transcriptional responses to temperature and light stress, integrating comparative transcriptomics with promoter analysis to explore potential co-expression with bZIP factors. A total of 48 *CcGRAS* genes were identified and found to be unevenly distributed across nine chromosomes. Expression profiling revealed that *CcGRAS* genes are markedly more responsive to varying light intensities (476, 8340 lx) than to temperature stresses (15, 35 °C), relative to controls (2060 lx for light, 25 °C for temperature). Co-expression analysis uncovered an underground tissue-specific module in which *CcbZIP16* is upregulated with four *CcGRAS* genes (*CcGRAS11*, *CcGRAS12*, *CcGRAS43*, *CcGRAS48*) that are coordinately upregulated specifically under low-light conditions. The promoters of these co-expressed genes are significantly enriched in canonical light-responsive cis-elements, providing correlative evidence for their coordinated transcriptional control. Together, these findings identify a tissue-specific *GRAS-bZIP* co-expressed gene set under light stress and suggest a candidate regulatory framework for dissecting light adaptation mechanisms. This work also provides a foundation for targeted genetic improvements in stress tolerance and alkaloid biosynthesis in this important medicinal plant.

## 1. Introduction

Transcription factors (TFs) are essential components of plant signaling pathways and play critical regulatory roles in stress responses. They bind specifically to cis-acting elements, thereby repressing or activating transcription [1]. With the rapid advancement of high-throughput sequencing technologies, whole-genome sequencing has been completed for numerous plant species, leading to the identification of many TFs involved in growth regulation and stress responses, including drought, high salinity, and low temperature. Examples include NAC (NAM, ATAF, and CUC) [2], MYB (v-myb avian myeloblastosis viral oncogene homolog) [3], DREB/ERF (dehydration-responsive element binding/ethylene-responsive factor) [4], WRKY (WRKY domain) [5], and GRAS (GAI, RGA, SCR) transcription factors [6].

GRAS proteins typically range from 400 to 770 amino acids and share a modular structure consisting of a variable N-terminal region and a highly conserved C-terminal domain. The N-terminus often contains an intrinsically disordered region (IDR) that facilitates protein–protein interactions, while the C-terminus harbors several conserved motifs—including leucine heptad repeats (LHR I and II), the VHIID domain, PFYRE, and SAW—that are critical for DNA binding and dimerization [7,8]. Based on phylogenetic relationships and domain architecture, GRAS family members are classified into multiple subfamilies, including LISCL, DELLA, PAT1, HAM, LS, SCR, SHR, and SCL3 [9,10]. Genome-wide characterization of GRAS genes has been performed in numerous species, such as *Arabidopsis thaliana* [11], *Oryza sativa* [12], *Glycine max* [13], and *Gossypium hirsutum* [14], revealing both evolutionary conservation and lineage-specific expansion of this family.

Functionally, GRAS TFs are best known for their roles in plant development. For instance, members of the DELLA subfamily act as negative regulators of gibberellin signaling, controlling seed germination, stem elongation, and flowering time [15,16]. Beyond development, accumulating evidence points to their involvement in abiotic stress responses. In *Populus davidiana × P. bolleana*, *PdbSCL1* was involved in stress response and development [6]. In *Capsicum annuum*, CaGIR1 was involved in negatively regulating the ABA signaling pathway and drought stress response by mediating the degradation of the CaGRAS1 protein [17]. These examples highlight the functional diversity of GRAS TFs in mediating environmental signals.

*Coptis chinensis* is a valuable medicinal plant whose rhizomes produce berberine, a bioactive alkaloid with broad pharmacological activities that serves as a key quality indicator of this species [18]. Light intensity is a critical regulator of both plant development and the biosynthesis of medicinal compounds in medicinal plants [19]. However, the molecular mechanisms by which *C. chinensis* perceives and responds to light stress remain poorly understood. Specifically, the GRAS family in this species has not been systematically characterized, and its potential functional interaction with other light-responsive TF families, such as bZIPs, has not been explored.

In this study, we aimed to characterize the GRAS transcription factor family in *C. chinensis* and investigate its potential co-expression with bZIP factors under abiotic stress. Specifically, we addressed the following questions: (1) How many GRAS genes are present in the *C. chinensis* genome, and what are their evolutionary characteristics? (2) Do *CcGRAS* genes exhibit tissue-specific and stress-type-dependent expression patterns? (3) Is there evidence for co-expression between *CcGRAS* and *CcbZIP* genes under light stress, and what cis-elements may coordinate such expression? To answer these questions, we performed genome-wide identification, integrating our data with previously published *CcbZIP* genes [20]. This study provides the first comprehensive characterization of the GRAS family in *C. chinensis* and offers new insights into light stress responses in this medicinal plant.

## 2. Results

### 2.1. Genome-Wide Identification, Classification and Phylogenetic Analysis of CcGRAS Genes

A total of 48 non-redundant CcGRAS proteins with complete GRAS conserved domains were identified and systematically renamed as CcGRAS01 to CcGRAS48 according to their chromosomal positions (Table 1). Physicochemical analysis revealed considerable diversity among CcGRAS proteins: protein length ranged from 126 (CcGRAS35) to 869 (CcGRAS33) amino acids, with corresponding molecular weights of 13.74–95.81 kDa. The majority of CcGRAS proteins exhibited slightly acidic isoelectric points (pI 5.0–6.0) and were predicted to be hydrophilic, as indicated by negative grand average of hydropathy (GRAVY) values for most members. Subcellular localization prediction suggested that most CcGRAS proteins are nuclear-localized, consistent with their role as transcription factors, while a small number were predicted to localize to other organelles, implying potential non-nuclear functions (Table 1).

**Figure 1:**
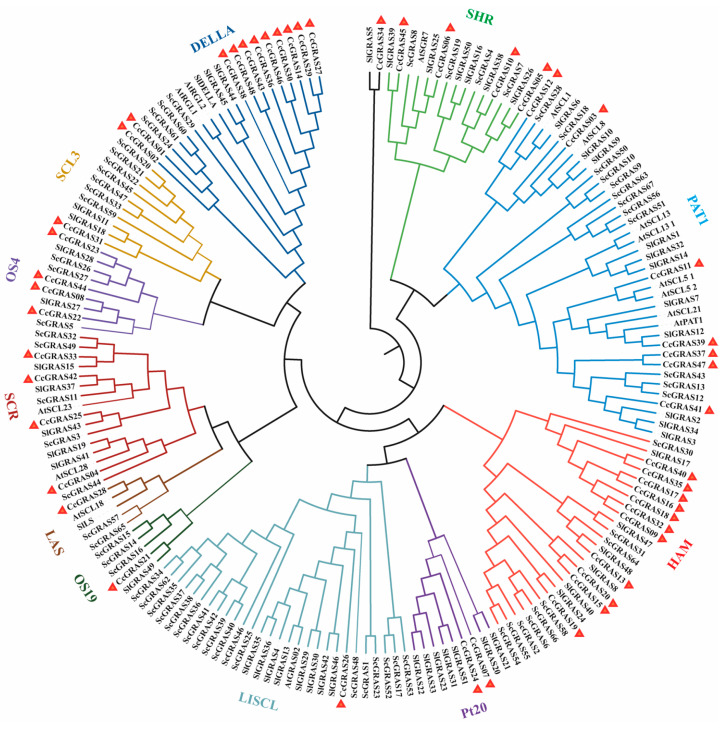
Phylogenetic tree of the GRAS gene family in *C. chinensis* and other plants. The maximum likelihood (ML) phylogenetic tree was constructed using MEGA7.0. Subfamilies are indicated by different colored arcs. Bootstrap values from 1000 replicates are shown at key nodes. The red triangle symbol indicates candidate target genes.

To elucidate the evolutionary relationships of CcGRAS proteins, a maximum-likelihood phylogenetic tree was constructed using full-length amino acid sequences of 48 CcGRAS proteins together with GRAS orthologs from other plants (Figure 1). Based on clustering with known *Arabidopsis thaliana*, *Solanum lycopersicum*, and *Secale cereale*, the 48 CcGRAS proteins were classified into 11 subfamilies (Figure 1). The amino acid sequence of all genes was listed in Table S1. The DELLA and HAM subfamilies were the largest, each containing 11 members (22.9% of the family), followed by the PAT1 subfamily with 7 members (14.6%). In contrast, five subfamilies—SCL3, LAS, OS19, LISCL, and CcGRAS34—contained only one member each, reflecting uneven expansion and functional diversification within the CcGRAS family during evolution.

### 2.2. Chromosomal Distribution of CcGRAS

**Figure 2:**
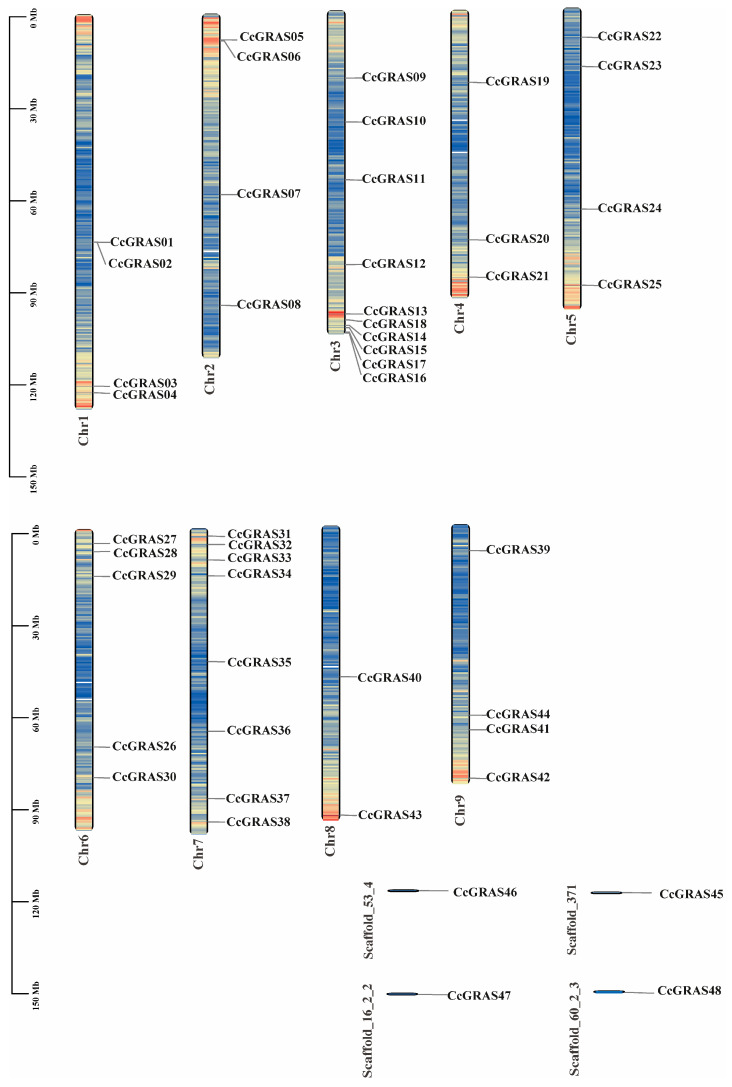
Chromosomal localization of CcGRAS genes in *C. chinensis*.

To investigate the genomic distribution of the *CcGRAS* gene family in *C. chinensis*, we performed chromosome localization analysis. As shown in Figure 2, 48 *CcGRAS* genes were mapped to 9 chromosomes (Chr1–Chr9) and 4 scaffolds, with an uneven distribution across the genome. Chromosome 3 contained the highest number of *CcGRAS* genes (8), followed by Chr7 (7), while Chr9 contained only 2.

Notably, several *CcGRAS* genes were organized in close proximity as adjacent gene pairs on the same chromosomes, including CcGRAS01/02 on Chr1, CcGRAS05/06 on Chr2, and CcGRAS16/17 on Chr3. These clustered arrangements suggest that tandem duplication may have contributed to the expansion of the *CcGRAS* family in *C. chinensis*. In addition, a dense cluster of multiple genes was observed on Chr3 (CcGRAS11–18), further supporting the occurrence of local duplication events. Four *CcGRAS* genes were located on unanchored scaffolds, indicating that the current genome assembly of *C. chinensis* remains incomplete. This limitation may lead to an underestimation of total GRAS family members and affect the accuracy of chromosomal distribution and synteny analyses. Future improvements in genome assembly quality will be necessary to fully resolve the genomic organization of the *CcGRAS* family.

### 2.3. Gene Motif and Conserved Domain Analysis

**Figure 3:**
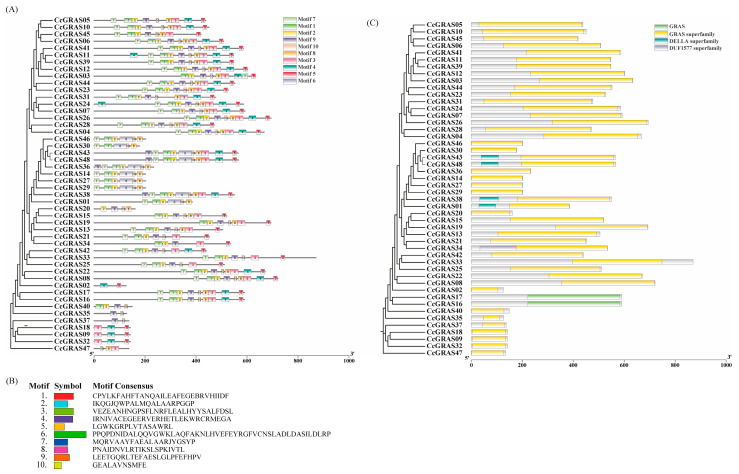
Conserved motif and domain analysis of CcGRAS proteins in *C. chinensis*. (**A**) Phylogenetic tree of CcGRAS proteins with the distribution of 10 conserved motifs (Motif 1–10) indicated by colored boxes. (**B**) Consensus sequences of the 10 conserved motifs identified by MEME analysis. (**C**) Conserved domain architecture of CcGRAS proteins predicted. The canonical GRAS domain is shown in yellow, while other domains (DELLA, DUF1577) are highlighted in distinct colors.

To characterize the structural features and evolutionary relationships of the *CcGRAS* gene family in *C. chinensis*, we performed a phylogenetic analysis combined with conserved motif and domain prediction. As shown in Figure 3A, MEME analysis identified 10 distinct conserved motifs (Motif 1–10) among the 48 CcGRAS proteins. Most CcGRAS proteins contained a similar set of core motifs, including Motif 1, Motif 2, and Motif 6, which are characteristic of the GRAS family. The consensus sequences of the 10 identified motifs are provided in Figure 3B, confirming their conservation across the CcGRAS family. However, subtle variations in motif composition were observed among different phylogenetic clades, suggesting functional diversification within the family.

Consistent with the motif analysis, NCBI Conserved Domain Database (CDD) prediction revealed that all 48 CcGRAS proteins contained the canonical GRAS conserved domain (Figure 3C). Additionally, a subset of proteins was found to contain other conserved domains, such as the DELLA domain (associated with gibberellin signaling) and the DUF1577 domain, which further supports the functional specialization of different CcGRAS subfamilies.

### 2.4. Expression Patterns of CcGRAS Genes Under Different Temperature Conditions

**Figure 4:**
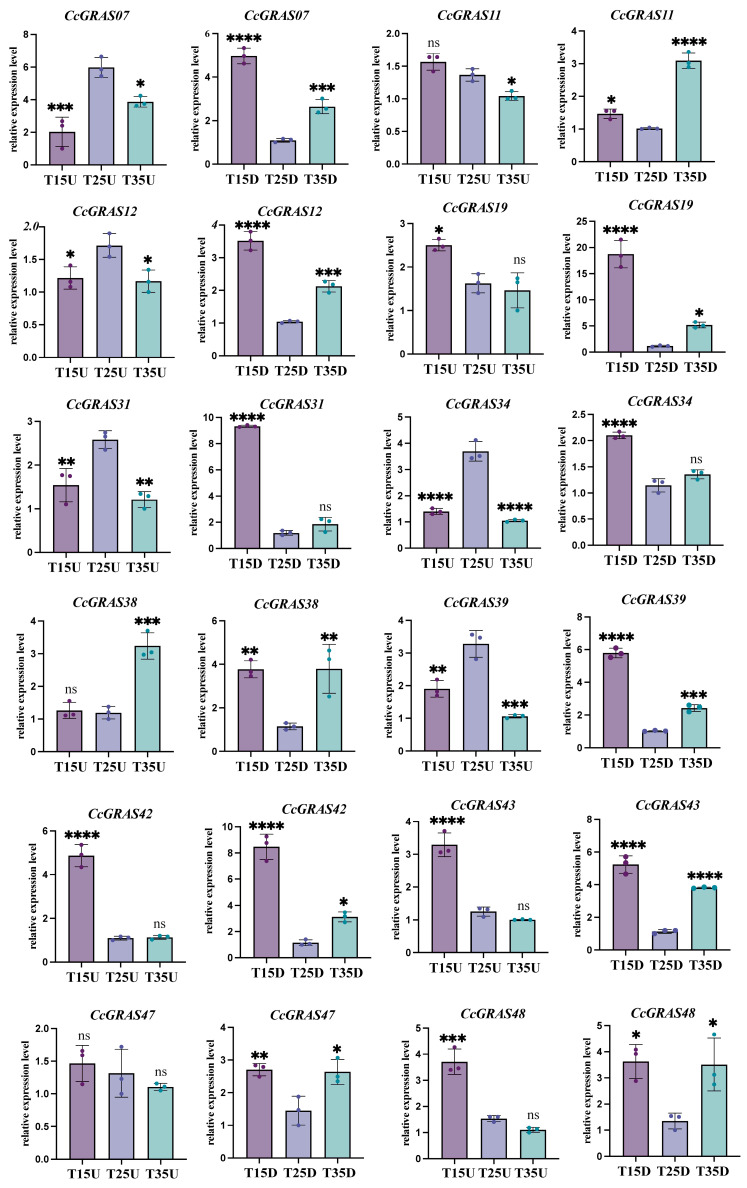
Expression patterns of selected *CcGRAS* genes under different temperature conditions. qRT-PCR analysis of *CcGRAS07*, *CcGRAS11*, *CcGRAS12*, *CcGRAS19*, *CcGRAS31*, *CcGRAS34*, *CcGRAS38*, *CcGRAS39*, *CcGRAS42*, *CcGRAS43*, *CcGRAS47* and *CcGRAS48* in aboveground (**left**) and underground (**right**) parts. Data are presented as mean ± SD (n = 3). Asterisks indicate significant differences compared to the control (25 °C) (* *p* < 0.05; ** *p* < 0.005; *** *p* < 0.0005; **** *p* < 0.0001). ns stands for no significant difference.

To assess the involvement of *CcGRAS* genes in thermal stress responses, we examined their expression in aboveground (U) and underground (D) parts under low (15 °C, T15), control (25 °C, T25), and high (35 °C, T35) temperature conditions using qRT-PCR (Figure 4). The 12 *CcGRAS* genes analyzed were selected based on their most pronounced expression changes in transcriptomic data. All primer sequences are listed in Table S2.

In the aboveground parts, temperature stress elicited a predominantly repressive response, with the direction and magnitude of regulation depending on the specific stress. Under low temperature (15 °C), *CcGRAS19*, *CcGRAS31*, *CcGRAS42*, *CcGRAS43*, and *CcGRAS48* were significantly induced (1.5- to 4.5-fold), whereas *CcGRAS07*, *CcGRAS12*, *CcGRAS34*, and *CcGRAS39* were downregulated (1.4- to 2.9-fold). Under high temperature (35 °C), *CcGRAS11*, *CcGRAS12*, *CcGRAS31*, *CcGRAS34*, and *CcGRAS39* showed reduced expression (1.3- to 3.5-fold), and only *CcGRAS38* was markedly induced (2.7-fold).

In contrast, the underground parts exhibited predominantly upregulation of *CcGRAS* genes under both temperature stresses. Low temperature induced nearly all tested genes, with *CcGRAS19* showing the strongest response (16.3-fold), followed by *CcGRAS31* (7.8-fold), *CcGRAS42* (7.4-fold), and *CcGRAS39* (5.7-fold). High temperature also triggered upregulation of a distinct set of genes (*CcGRAS11*, *CcGRAS19*, *CcGRAS38*, *CcGRAS42*, *CcGRAS43*, *CcGRAS48*) with fold changes ranging from 2.6 to 4.5. Notably, *CcGRAS31* and *CcGRAS34* were cold-specific responders, showing no significant change under heat stress.

Together, these results indicate that *CcGRAS* genes are responsive to temperature stress but exhibit clear tissue- and stress-dependent expression patterns. The pronounced upregulation in underground tissues under low temperature is consistent with the ecological preference of *C. chinensis* for cool environments, suggesting a potential role for these genes in cold adaptation.

### 2.5. Expression Patterns of CcGRAS Genes Under Different Light Conditions

**Figure 5:**
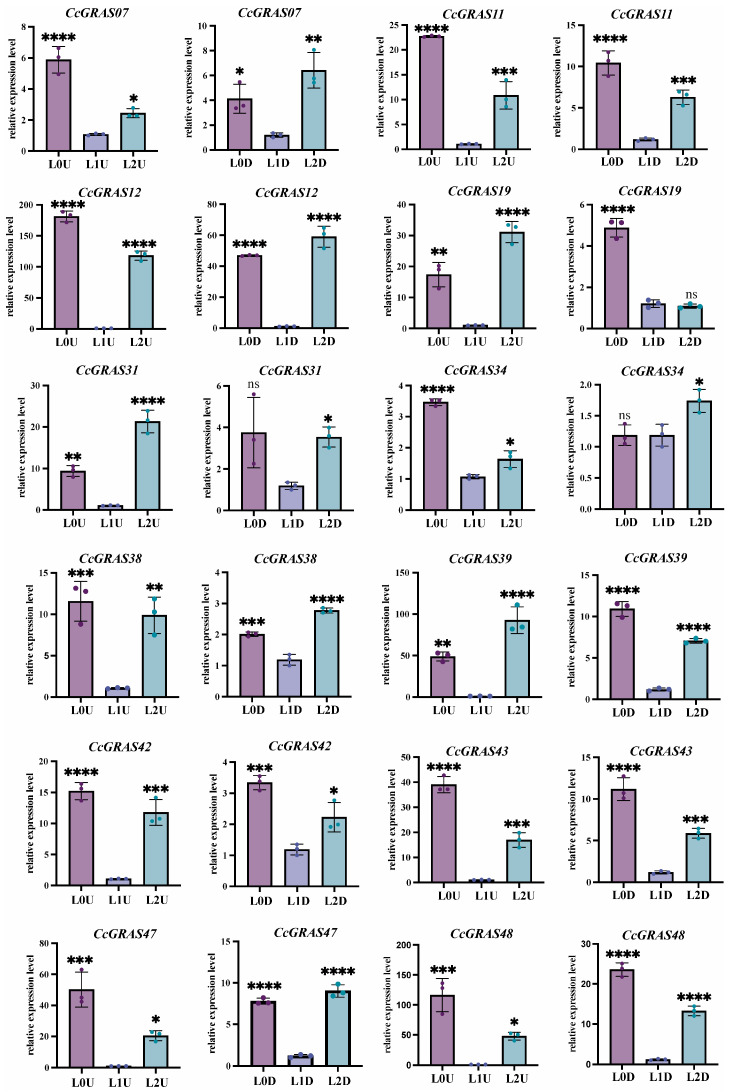
Expression patterns of selected *CcGRAS* genes under different light conditions. qRT-PCR analysis of *CcGRAS07*, *CcGRAS11*, *CcGRAS12*, *CcGRAS19*, *CcGRAS31*, *CcGRAS34*, *CcGRAS38*, *CcGRAS39*, *CcGRAS42*, *CcGRAS43*, *CcGRAS47* and *CcGRAS48* in aboveground (**left**) and underground (**right**) parts. Data are presented as mean ± SD (n = 3). Asterisks indicate significant differences compared to the control (25 °C) at the same time point (* *p* < 0.05; ** *p* < 0.005; *** *p* < 0.0005; **** *p* < 0.0001). ns stands for no significant difference.

Light stress elicited strong and widespread upregulation of *CcGRAS* genes in aboveground parts, with most tested genes showing significant induction under both low (476 lx) and high (8340 lx) light conditions relative to the control (2060 lx) (Figure 5). Among these, *CcGRAS43*, *CcGRAS11*, and *CcGRAS12* were the most highly responsive, with expression increases ranging from 36.8- to 110-fold. A second group of genes, including *CcGRAS39*, *CcGRAS42*, and *CcGRAS47*, showed moderate to strong induction (9- to 82-fold), whereas *CcGRAS07* and *CcGRAS31* exhibited only modest upregulation (less than 5-fold).

In underground parts, the majority of *CcGRAS* genes were also upregulated under light stress, albeit with generally lower fold changes compared to aboveground tissues. *CcGRAS11* and *CcGRAS43* remained the most responsive, with robust induction under both low and high light. Interestingly, *CcGRAS12* displayed a distinct tissue-dependent expression pattern: it was preferentially induced under low light in aboveground parts, but under high light in underground parts. This contrasting behavior suggests that *CcGRAS12* may integrate tissue-specific light signals. Collectively, the widespread transcriptional activation of *CcGRAS* genes across both tissue types supports their involvement in light signal transduction in *C. chinensis*.

### 2.6. Comparative Stress Responsiveness of CcGRAS and CcbZIP Families

**Figure 6:**
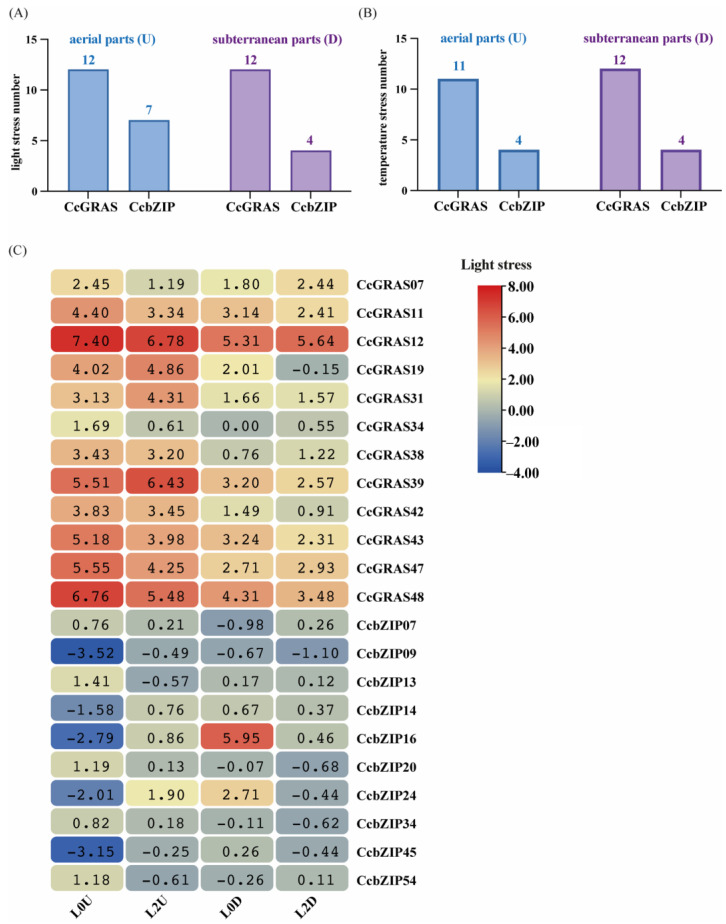
Stress response profiles of *CcGRAS* and *CcbZIP* gene families in *C. chinensis* under light and temperature stress. (**A**) Number of *CcGRAS* and *CcbZIP* genes with significant expression responses to light stress in aboveground (U) and underground (D) parts. (**B**) Number of *CcGRAS* and *CcbZIP* genes with significant expression responses to temperature stress in aboveground (U) and underground (D) parts. (**C**) Circular heatmap showing the log_2_-transformed fold change (log_2_FC) values of *CcGRAS* and *CcbZIP* genes under different light treatments (L0U, L2U, L0D, L2D) relative to the corresponding control (L1U for aboveground parts, L1D for underground parts). The color scale represents the magnitude of log_2_FC, with red indicating upregulation and blue indicating downregulation relative to the control.

To compare the stress response profiles of the *CcGRAS* and *CcbZIP* gene families, we quantified the number of genes significantly responsive to light and temperature stress in the aboveground and underground parts. Under light stress (Figure 6A), 12 *CcGRAS* genes were responsive in both aboveground and underground parts, whereas the number of responsive *CcbZIP* genes was higher in aboveground (7 genes) than in underground (4 genes) tissues. Under temperature stress (Figure 6B), the total number of responsive genes was lower for both families: 11–12 *CcGRAS* genes and 4 *CcbZIP* genes responded across tissue types. Notably, although the number of responsive *CcGRAS* genes was similar between light and temperature stress, the magnitude of their expression changes was substantially greater under light stress (Figure 4 vs. Figure 5), indicating a stronger transcriptional response to light signals.

To further examine the expression dynamics of these responsive genes under light stress, we constructed a circular heatmap of log_2_-transformed fold change (log_2_FC) values (Figure 6C). This analysis identified a subset of genes (*CcGRAS11*, *CcGRAS12*, *CcGRAS39*, *CcGRAS43*, *CcGRAS48*, *CcbZIP16*, and *CcbZIP24*) with the most pronounced expression changes across light treatments. These genes exhibited correlated expression patterns, with coordinated upregulation particularly evident in underground parts under low-light (L0D). This observation defines a candidate co-expressed gene set potentially involved in light stress responses. However, co-expression alone does not establish direct regulatory interactions (e.g., protein-protein or protein-DNA binding); experimental validation is required to determine whether these genes function as a coherent regulatory module [7].

### 2.7. Co-Expression Analysis of CcGRAS and CcbZIP Gene Pairs Under Light Stress

**Figure 7:**
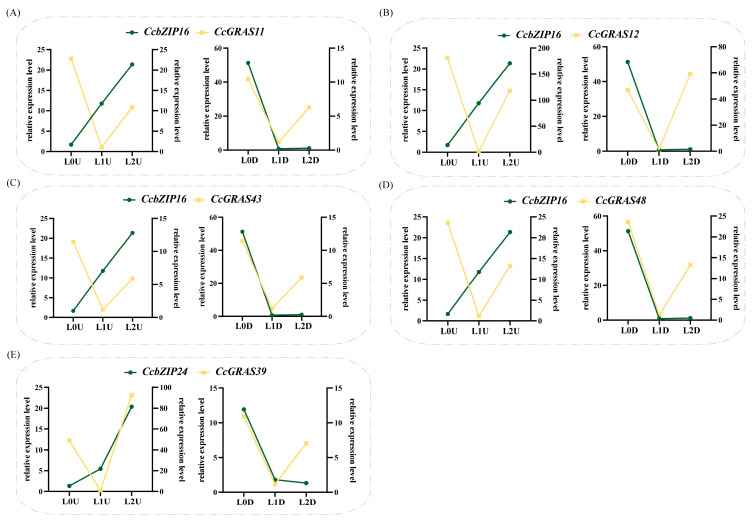
Expression patterns of representative *CcGRAS* and *CcbZIP* gene pairs in aboveground and underground parts of *C. chinensis* under different light intensities. (**A**) Expression profiles of *CcbZIP16* and *CcGRAS11*. (**B**) Expression profiles of *CcbZIP16* and *CcGRAS12*. (**C**) Expression profiles of *CcbZIP16* and *CcGRAS43*. (**D**) Expression profiles of *CcbZIP16* and *CcGRAS48*. (**E**) Expression profiles of *CcbZIP24* and *CcGRAS39*. Green lines indicate the relative expression levels of *CcbZIP* genes, and orange lines indicate the relative expression levels of *CcGRAS* genes. L0U and L2U represent low and high light intensities in the aboveground parts, respectively. L0D and L2D represent low and high light intensities in the underground parts, respectively. L1U and L1D represent the corresponding control light intensities.

To identify potential co-expression relationships between *CcGRAS* and *CcbZIP* transcription factors under light stress, we calculated Pearson correlation coefficients for all pairwise combinations of these genes using their expression data under light treatments (Table S3). As shown in Figure 7, *CcbZIP16* exhibited a highly similar expression trend with four *CcGRAS* genes (*CcGRAS11*, *CcGRAS12*, *CcGRAS43*, and *CcGRAS48*) in underground parts. All five genes were significantly upregulated under low light (L0D) relative to the control (L1D) and remained elevated under high light (L2D). In the aboveground parts, by contrast, their responses diverged under low light (L0U): *CcbZIP16* was downregulated, whereas the four *CcGRAS* genes were induced. This tissue-dependent divergence suggests that the *CcbZIP16–CcGRAS* regulatory relationship may be context-specific.

The four *CcGRAS* genes co-expressed with *CcbZIP16* displayed subtle but distinct expression characteristics. *CcGRAS11*, *CcGRAS43*, and *CcGRAS48* were induced by both low and high light in aboveground and underground parts, with expression levels under low light consistently higher than those under high light across both tissue types. In contrast, *CcGRAS12* exhibited a distinct tissue-dependent pattern. In the aboveground parts, it was more strongly induced under low light than under high light; in the underground parts, the opposite was true, with higher expression under high light (Figure 7B). This unique response suggests that *CcGRAS12* may be subject to additional tissue-specific regulatory inputs.

The *CcGRAS39–CcbZIP24* pair also showed tissue- and light-dependent expression patterns. In the aboveground parts, *CcbZIP24* was downregulated under low light but upregulated under high light, whereas *CcGRAS39* was induced under both conditions, with stronger induction under high light. In underground parts, however, their expression diverged: *CcbZIP24* was downregulated, while *CcGRAS39* remained upregulated (Figure 7E).

Collectively, these results identify a conserved co-expression module involving *CcbZIP16* and multiple *CcGRAS* genes in underground parts under light stress, particularly under low-light conditions. However, co-expression alone does not demonstrate direct regulatory interactions (e.g., protein–protein or protein–DNA binding), and the functional relevance of these observed patterns requires experimental validation.

### 2.8. Cis-Acting Element Analysis of Promoters Co-Expressed CcGRAS and CcbZIP Genes

**Figure 8:**
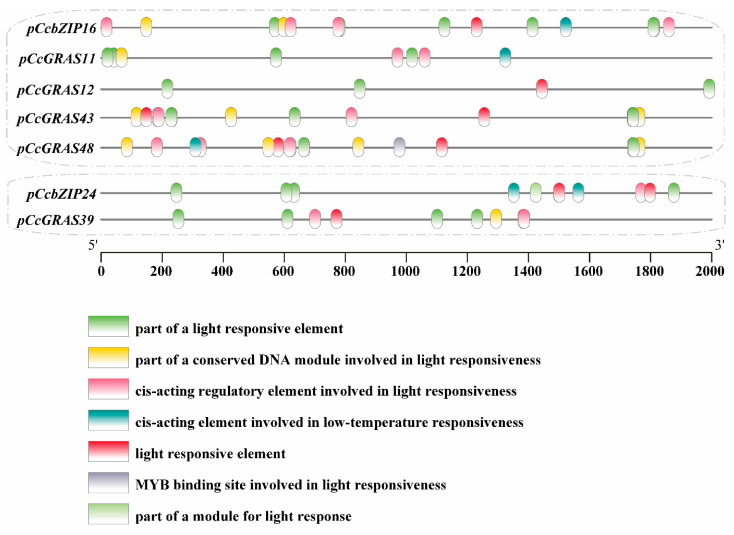
Cis-acting element analysis of the promoters of co-regulated *CcGRAS* and *CcbZIP* gene pairs in *C. chinensis*. The 2000 bp promoter regions upstream of the translation start site (ATG) of *CcbZIP16*, *CcGRAS11*, *CcGRAS12*, *CcGRAS43*, *CcGRAS48*, *CcbZIP24*, and *CcGRAS39* were analyzed using PlantCARE (https://bioinformatics.psb.ugent.be/webtools/plantcare/html/, accessed on 20 March 2026). Different colored boxes represent distinct types of cis-acting elements, as indicated in the legend. The scale at the bottom indicates the distance from the translation start site in base pairs (bp).

To investigate the molecular mechanisms underlying the co-expression of *CcGRAS* and *CcbZIP* genes under light stress, we analyzed the 2000 bp promoter regions upstream of the translation start site (ATG) for five core gene pairs (*CcbZIP16–CcGRAS11*, *CcbZIP16–CcGRAS12*, *CcbZIP16–CcGRAS43*, *CcbZIP16–CcGRAS48*, and *CcbZIP24–CcGRAS39*) using the PlantCARE database. As shown in Figure 8, all promoters were highly enriched in cis-elements, including G-box, GT1-motif, and I-box (complete element annotations are provided in Table S4).

The promoters of *CcbZIP16* and its co-expressed *CcGRAS* genes (*CcGRAS11*, *CcGRAS12*, *CcGRAS43*, and *CcGRAS48*) shared a similar complement of light-responsive elements. Specifically, multiple G-box copies were present in the promoters of *CcbZIP16*, *CcGRAS11*, and *CcGRAS43* (Figure 8, green boxes), consistent with their strong induction under low light. For the *CcbZIP24–CcGRAS39* pair, both promoters contained light-responsive elements in common, including part of a light-responsive element. In addition, each promoter possessed unique elements: the *CcGRAS39* promoter contained part of a conserved DNA module involved in light responsiveness, whereas the *CcbZIP24* promoter contained a cis-acting element involved in low-temperature responsiveness (Figure 8). These differences in promoter composition may relate to the divergent expression patterns observed for this gene pair under certain conditions.

It should be noted that G-box and related light-responsive elements are commonly found in plant promoters; therefore, their presence alone does not demonstrate direct regulatory interactions. Instead, these promoter analyses provide correlative evidence consistent with a potential role for these genes in light-responsive transcriptional regulation.

## 3. Discussion

Transcription factors of the GRAS family are well-documented for their diverse roles in plant growth, development, and abiotic stress responses across various species [21]. However, their systematic characterization in *C. chinensis*, a medically important herb with unique ecological and metabolic characteristics, has remained unaddressed prior to this study. Our genome-wide identification of 48 *CcGRAS* genes, classified into 11 distinct subfamilies, provides the first comprehensive insights into the GRAS family in this species. Among them, the SCL3, LAS, OS19, and LISCL subfamilies each contain only one member, reflecting functional specialization and evolutionary conservation of these subfamilies in *C. chinensis*.

One observation of this work is the pronounced responsiveness of the *CcGRAS* family to light stress, which far exceeded its response to temperature fluctuations. This raises the possibility that GRAS TFs in *C. chinensis* may play a role as integrators of light signals, a function that may be particularly relevant for a plant whose economic value is tied to light-regulated alkaloid accumulation in underground rhizomes [19,22]. The tissue-specific expression patterns we observed further support this hypothesis, with *CcGRAS43* showing dramatic induction in aboveground parts, while *CcGRAS12* displayed a more nuanced, tissue-dependent response to light intensity.

Another notable observation is the tissue-specific co-expression module involving *CcbZIP16* and four *CcGRAS* genes (*11*/*12*/*43*/*48*) in underground parts under low light. bZIP TFs, particularly those containing G-box binding domains, are central to light signaling pathways [23]. The synchronous upregulation of this module in the rhizome—the primary site of berberine biosynthesis—under low-light conditions is particularly intriguing. It suggests a potential mechanism by which the plant fine-tunes underground growth and/or secondary metabolism in response to aboveground light conditions. This hypothesis is further supported by the enrichment of light-responsive cis-elements (G-box, GT1-motif) in the promoters of all five genes, providing correlative evidence for their coordinated transcriptional control, potentially via shared upstream regulators like HY5 [24]. Furthermore, physical and genetic interactions between *GRAS* and *bZIP* transcription factors have been increasingly recognized as important regulatory modules in plants. For instance, the Arabidopsis GRAS protein SCL14 interacts with class II TGA factors (bZIP family) to activate stress-inducible promoters [25]. Consistent with our findings, co-expression analyses in other species have also revealed close regulatory relationships between *GRAS* and *bZIP* gene families under stress conditions [7]. These precedents raise the possibility that *CcbZIP16* may directly interact with its co-expressed *CcGRAS* partners, forming a regulatory complex that integrates light signals in underground parts. However, this hypothesis remains to be validated by protein–protein interaction and DNA-binding studies.

The functional divergence within this module, exemplified by CcGRAS12’s unique response profile, may be attributed to subtle variations in its promoter architecture. The presence of a unique combination of light-responsive elements could allow for differential binding of transcription factors under varying light intensities or in different tissue contexts, leading to its specialized expression pattern [26]. Similarly, the presence of low-temperature response elements in the *CcbZIP24* promoter may explain its expression divergence from *CcGRAS39* under certain conditions, highlighting how individual gene promoters integrate multiple signals.

The consistent upregulation of *CcGRAS* genes in underground tissues under light stress may reflect systemic signaling from aerial parts. Light perception by aboveground tissues likely triggers systemic signals (e.g., hormones, reactive oxygen species, or electrical signals) that propagate to the rhizome, where GRAS and bZIP factors act as downstream integrators. This hypothesis is consistent with previous reports of light-induced systemic responses in other plant species [27].

Several limitations should be considered when interpreting our findings. First, this study is primarily descriptive and correlative. While we identified co-expression patterns between *CcGRAS* and *CcbZIP* genes under light stress, we did not perform functional validation of their regulatory relationships. Second, the presence of light-responsive cis-elements in promoter regions is suggestive but not conclusive evidence of direct regulation, as such motifs are commonly found in plant promoters. Third, the corrected expression fold changes are biologically plausible but still await independent validation. Fourth, while our findings suggest a potential link between the *CcbZIP16–CcGRAS* module and berberine biosynthesis, direct evidence (e.g., metabolite quantification) is lacking. Previous studies have shown that light intensity affects berberine accumulation in *C. chinensis* rhizomes [20], but causal relationships with specific transcription factors remain to be established. Therefore, the proposed GRAS–bZIP regulatory relationships remain hypothetical until tested experimentally.

Future studies employing techniques such as yeast two-hybrid (Y2H) assays, bimolecular fluorescence complementation (BiFC), and chromatin immunoprecipitation sequencing (ChIP-seq) will be crucial to elucidate the molecular architecture of this network. Furthermore, functional characterization of the core genes (*CcbZIP16*, *CcGRAS11/43/48*) through overexpression or CRISPR/Cas9-mediated knockout in *C. chinensis* will be essential to determine their precise roles in light stress tolerance and the regulation of berberine biosynthesis. This study lays a critical foundation for such work, opening new avenues for molecular breeding strategies aimed at improving the stress resilience and medicinal quality of this important herb.

## 4. Materials and Methods

### 4.1. Plant Materials and Growth Conditions

Two-year-old *Coptis chinensis* Franch (Ranunculaceae) plants were obtained from the cultivation base in Shizhu County, Chongqing, China. Voucher specimens were deposited at the Chongqing Academy of Chinese Materia Medica after taxonomic authentication by Prof. Yuan Pan. Uniformly grown, disease- and pest-free plants were selected [18] and transplanted into soil in growth chambers under controlled conditions: 26 °C (light period)/20 °C (dark period), 60% relative humidity, and a 16 h light/8 h dark photoperiod. After a three-week acclimation period, uniformly developed seedlings were subjected to stress treatments.

All stress treatments lasted for 48 h. For the temperature stress, seedlings were moved to growth chambers set at 15 °C (low temperature), 25 °C (control), or 35 °C (high temperature), with light intensity and photoperiod unchanged from acclimation conditions. For the light stress treatments, white LED lights were used to deliver three intensities measured at canopy height: 476 lx (low light, L0), 2060 lx (normal light, L1), and 8340 lx (high light, L2), with temperature maintained at 25 °C and the photoperiod unchanged. After treatment, aboveground (U) and underground (D) parts were harvested separately, immediately flash-frozen in liquid nitrogen, and stored at −80 °C for subsequent RNA extraction. Three biological replicates were prepared, each consisting of a composite sample from three individual seedlings [20].

### 4.2. Identification of GRAS Family Members

To identify GRAS family members in *C. chinensis*, a hidden Markov model (HMM) search was performed. The HMM profile of the GRAS conserved domain (PF03514) was downloaded from the Pfam database and used as a query to search against the *C. chinensis* protein database (BioProject: SAMN15658057) using HMMER 3.0 software, with an E-value threshold of 1 × 10^−5^. All candidate sequences obtained were then verified for the presence of the complete GRAS conserved domain using the NCBI Conserved Domain Database (CDD). The ExPASy ProtParam online tool (https://web.expasy.org/protparam/, accessed on 20 March 2026) was used to predict the physicochemical properties of the encoded proteins, including molecular weight (MW), theoretical isoelectric point (pI), and grand average of hydropathy (GRAVY). Subcellular localization was predicted using CELLO v2.5 [28].

### 4.3. Phylogenetic, Conserved Motif, Chromosomal Distribution Analysis

A Maximum Likelihood (ML) phylogenetic tree was constructed using MEGA7.0 with the JTT substitution model and 1000 bootstrap replicates. CcGRAS proteins were classified into subfamilies based on their clustering with known GRAS subfamilies from *Arabidopsis thaliana* [29], *Solanum lycopersicum* [10], and *Secale cereale* [30]. Conserved motifs were identified using MEME suite (https://meme-suite.org/meme/, accessed on 20 March 2026) with parameters set to a maximum of 10 motifs and an optimal motif width of 6–50 amino acids [31]. The distribution of conserved motifs and the chromosomal locations of *CcGRAS* genes were visualized using TBtools v2.323 [32].

### 4.4. RNA Extraction, Transcriptome Sequencing and qRT-PCR

Total RNA was extracted from approximately 100 mg of frozen tissue using TRIzol reagent (Invitrogen, Carlsbad, CA, USA) following the manufacturer’s instructions. Genomic DNA was removed by DNase I (Takara, Shiga Prefecture, Japan), and RNA was verified by 1.5% agarose gel electrophoresis. Transcriptome sequencing, cDNA library construction and Illumina high-throughput sequencing were completed by a professional biotechnology company. Detailed information regarding transcriptome sequencing and FPKM calculation has been described in our previous study [20].

For qRT-PCR validation, the first-strand cDNA was synthesized from 1 μg of total RNA using the PrimeScript™ RT reagent kit with gDNA Eraser (Takara, Shiga Prefecture, Japan). Gene-specific primers spanning exon–exon junctions were designed using Primer-BLAST (https://www.ncbi.nlm.nih.gov/tools/primer-blast, accessed on 5 March 2026), and primer specificity was confirmed by a single band on 1.5% agarose gel and a single peak in melt curve analysis. The *C. chinensis* actin gene was used as the internal reference [18]. qRT-PCR was performed in a 20 μL reaction system containing 10 μL of 2× qPCR MasterMix (Applied Biological Materials, Richmond, BC, Canada), 0.8 μL of each forward and reverse primer (10 μM), 2 μL of 1:10 diluted cDNA template, and 6.4 μL of nuclease-free water. The amplification was carried out on a CFX96 Touch Real-Time PCR Detection System (Bio-Rad, Hercules, CA, USA) with the thermal cycling program: 95 °C pre-denaturation for 30 s, followed by 40 cycles of 95 °C denaturation for 5 s and 60 °C annealing/extension for 30 s. A melt curve analysis (65 °C to 95 °C, with an increment of 0.5 °C per step) was performed after amplification to further confirm the specificity of the PCR products. All qRT-PCR reactions were performed with three biological replicates and three technical replicates for each sample. The 2^−ΔΔCt^ method was used to calculate the relative expression level of target genes, and Statistical significance was assessed by one-way ANOVA with Tukey’s HSD post hoc test using GraphPad Prism v10.0. Statistical significance was indicated as *p* < 0.05, *p* < 0.005, and * *p* < 0.0005 [33,34].

### 4.5. Co-Expression Analysis of CcGRAS and CcbZIP Genes Under Light Stress

Transcriptome and qRT-PCR expression data of *CcbZIP* genes under light stress were obtained from our previous study [20]. Pearson correlation coefficients between the expression levels of *CcGRAS* and *CcbZIP* genes were calculated using OmicShare (https://www.omicshare.com, accessed on 12 March 2026) [35]. Genes with an absolute correlation coefficient |r| ≥ 0.9 and *p* < 0.05 were defined as significantly co-expressed gene pairs. Co-expression heatmaps and tissue-specific expression patterns were visualized using TBtools v2.323 [32].

### 4.6. Cis-Acting Element Analysis of Promoters

The 2000 bp promoter regions upstream of the translation start site (ATG) of co-regulated gene pairs (*CcbZIP16–CcGRAS11*, *CcbZIP16–CcGRAS12*, *CcbZIP16–CcGRAS43*, *CcbZIP16–CcGRAS48*, and *CcbZIP24–CcGRAS39*) were extracted from the *C. chinensis* genome database. Cis-acting elements in these promoter regions were predicted using the PlantCARE database, and the types and distribution of light-responsive elements were analyzed and visualized using TBtools software (v2.323).

### 4.7. Data Archiving Statement

The sequencing data were deposited in the National Genomics Data Center Short Read Archive under accession number PRJCA059627.

## 5. Conclusions

In summary, this study provides the first comprehensive characterization of the GRAS transcription factor family in *C. chinensis.* We identified a tissue-specific co-expression module in which *CcbZIP16* and four *CcGRAS* genes (*CcGRAS11*, *CcGRAS12*, *CcGRAS43*, *CcGRAS48*) are coordinately upregulated in underground parts specifically under low-light conditions. The promoters of these co-expressed genes are significantly enriched in canonical light-responsive cis-elements, including the G-box. This enrichment provides correlative evidence consistent with the hypothesis that these genes share upstream regulatory inputs. Notably, the expression patterns of these genes diverged between aboveground and underground tissues, suggesting that tissue-specific regulatory mechanisms may govern their responses to light stress. Collectively, these findings expand our understanding of light signaling networks in medicinal plants. Furthermore, they identify candidate genes (*CcbZIP16*, *CcGRAS11*, *CcGRAS43*, and *CcGRAS48*) for future functional studies aimed at improving stress tolerance. Future studies are required to validate whether the observed co-expression reflects direct regulatory interactions (e.g., protein-protein or protein-DNA binding) and to dissect the biological functions of these core genes. Such investigations will be essential for translating these discoveries into practical applications in the cultivation and breeding of *C. chinensis*.

## Figures and Tables

**Table 1:** Characteristics of CcGRAS.

Gene Name	Gene ID	MW (kDa)	PI	Instability Index	Liphatic Index	GRAVY	Gene_Chr	Gene_Start	Gene_End	Protein_Length (aa)	Dmain_Start	Dmain_End	Subcellular Localization
*CcGRAS01*	evm.model.Scaffold_110.20	42.08	5.43	50.04	91.17	−0.094	Chr1	74,173,297	74,174,451	384	151	383	Cytoplasmic
*CcGRAS02*	evm.model.Scaffold_110.21	13.74	4.95	49.17	92.88	0.082	Chr1	74,174,522	74,174,899	125	1	105	Chloroplast
*CcGRAS03*	evm.model.Scaffold_1.51	70.28	6.59	50.58	73.11	−0.453	Chr1	121,125,052	121,127,239	633	264	632	Nuclear
*CcGRAS04*	evm.model.Scaffold_350.35	74.38	5.6	63.08	83.14	−0.33	Chr1	123,258,265	123,260,265	666	285	652	Nuclear
*CcGRAS05*	evm.model.Scaffold_72.41	48.71	5.67	47.13	94.44	−0.084	Chr2	8,279,854	8,281,167	437	31	434	Chloroplastic
*CcGRAS06*	evm.model.Scaffold_72.85	57.35	5.54	46.96	70.97	−0.455	Chr2	8624,401	8,626,331	507	127	506	Nuclear
*CcGRAS07*	evm.model.Scaffold_15.193	67.13	4.85	40.59	82.81	−0.22	Chr2	58,756,724	58,758,499	591	215	587	Nuclear
*CcGRAS08*	evm.model.Scaffold_83.143	80.3	6.06	53.77	79.12	−0.448	Chr2	94,883,089	94,885,675	720	355	719	Nuclear
*CcGRAS09*	evm.model.Scaffold_3.29	16.08	6.14	42.62	76.81	−0.392	Chr3	21,881,365	21,881,790	141	3	135	Nuclear
*CcGRAS10*	evm.model.Scaffold_166.23	50.17	6.02	45.88	92.09	−0.075	Chr3	36,154,277	36,155,629	450	45	441	Chloroplastic
*CcGRAS11*	evm.model.Scaffold_47.353	60.56	5.69	51.88	78.79	−0.312	Chr3	55,034,602	55,040,987	546	176	546	Nuclear
*CcGRAS12*	evm.model.Scaffold_249.22	67.07	5.25	47.27	79.68	−0.384	Chr3	82,690,599	82,694,129	601	232	601	Nuclear
*CcGRAS13*	evm.model.Scaffold_417.147	56.13	5.58	36.71	79.37	−0.311	Chr3	98,781,631	98,783,145	504	105	492	Nuclear
*CcGRAS14*	evm.model.Scaffold_4.409	22.63	5.54	35.76	96.62	−0.044	Chr3	102,495,958	102,496,563	201	1	199	Mitochondrial
*CcGRAS15*	evm.model.Scaffold_4.345	59.19	5.81	45.74	82.12	−0.267	Chr3	103,259,627	103,261,920	519	155	518	Nuclear
*CcGRAS16*	evm.model.Scaffold_124.535	64.87	5.06	52.18	82.33	−0.295	Chr3	104,654,474	104,656,934	589	217	583	Nuclear
*CcGRAS17*	evm.model.Scaffold_95.41	64.87	5.06	52.18	82.33	−0.295	Chr3	104,985,106	104,987,568	589	217	583	Nuclear
*CcGRAS18*	evm.model.Scaffold_423.115	16.01	5.72	44.33	79.57	−0.324	Chr3	100,745,227	100,745,652	141	3	135	Nuclear
*CcGRAS19*	evm.model.Scaffold_215.54	76.21	5.36	55.18	83.66	−0.231	Chr4	23,420,470	23,422,548	692	328	691	Nuclear
*CcGRAS20*	evm.model.Scaffold_109.827	18.1	5.45	37.3	101.81	0.281	Chr4	74,751,525	74,752,007	160	2	157	Cytoplasmic
*CcGRAS21*	evm.model.Scaffold_41.288	52.01	6.2	49.21	85.83	−0.395	Chr4	86,925,339	86,926,694	451	76	449	Nuclear
*CcGRAS22*	evm.model.Scaffold_139.32	74.87	5.45	56.11	77.06	−0.382	Chr5	9,448,375	9,451,750	670	305	669	Nuclear
*CcGRAS23*	evm.model.Scaffold_287.72	58.22	5.92	49.26	89.71	−0.094	Chr5	19,011,065	19,012,642	525	153	524	Cytoplasmic
*CcGRAS24*	evm.model.Scaffold_20.410	66.53	5.35	52.37	78.53	−0.283	Chr5	65,478,022	65,479,779	585	203	572	Nuclear
*CcGRAS25*	evm.model.Scaffold_150.52	57.04	6.15	38.86	78.8	−0.209	Chr5	90,330,843	90,332,375	510	150	505	Endoplasmic Reticulum
*CcGRAS26*	evm.model.Scaffold_48.189	78.87	5.71	48.45	78.37	−0.552	Chr5	93,683,609	93,685,666	693	317	688	Nuclear
*CcGRAS27*	evm.model.Scaffold_155.22	22.55	5.7	35.22	92.74	−0.092	Chr6	4,503,424	4,504,029	201	1	199	Mitochondrial
*CcGRAS28*	evm.model.Scaffold_372.11	52.63	5.78	52.68	92.77	−0.137	Chr6	7,149,637	7,151,049	470	56	469	Nuclear
*CcGRAS29*	evm.model.Scaffold_130.71	22.55	5.7	35.22	92.74	−0.092	Chr6	15,128,383	15,128,988	201	1	199	Mitochondrial
*CcGRAS30*	evm.model.Scaffold_29.27	19.94	5.7	35.22	92.74	−0.092	Chr6	80,782,585	80,783,190	177	75	175	Mitochondrial
*CcGRAS31*	evm.model.Scaffold_264.209	53.32	5.86	53.69	95.11	−0.128	Chr7	2,411,989	2,414,094	476	50	471	Nuclear
*CcGRAS32*	evm.model.Scaffold_384.4	16.08	6.14	42.62	76.81	−0.392	Chr7	5,190,219	5,190,644	141	3	135	Nuclear
*CcGRAS33*	evm.model.Scaffold_273.165	95.81	6.37	50.13	84.3	−0.229	Chr7	10,188,077	10,191,951	869	392	748	Nuclear
*CcGRAS34*	evm.model.Scaffold_167.169	60.96	4.78	54.27	79.17	−0.274	Chr7	15,295,410	15,297,194	533	169	533	Chloroplastic
*CcGRAS35*	evm.model.Scaffold_85.142	13.88	5.5	41.02	104.44	0.356	Chr7	43,239,848	43,242,352	126	37	113	Cytoplasmic
*CcGRAS36*	evm.model.Scaffold_214.33	25.93	5.78	34.14	90.94	−0.103	Chr7	65,972,766	65,973,467	233	1	231	Cytoplasmic
*CcGRAS37*	evm.model.Scaffold_49.137	15.12	5.69	44.75	91.03	−0.118	Chr7	87,926,744	87,927,891	136	45	132	Cytoplasmic
*CcGRAS38*	evm.model.Scaffold_75.156	60.1	5.14	52.18	88.83	−0.138	Chr7	95,509,968	95,513,124	549	182	542	Nuclear
*CcGRAS39*	evm.model.Scaffold_149.78	61.29	5.9	53.54	83.24	−0.371	Chr9	8,405,855	8,411,287	547	177	547	Nuclear
*CcGRAS40*	evm.model.Scaffold_222.85	16.53	4.89	52.44	113.38	0.251	Chr8	48,966,241	48,967,535	148	2	129	Cytoplasmic
*CcGRAS41*	evm.model.Scaffold_180.17	65.45	5.15	51.93	74.2	−0.49	Chr9	66,737,034	66,738,788	584	215	583	Nuclear
*CcGRAS42*	evm.model.Scaffold_103.112	48.98	5.46	49.51	98.13	−0.167	Chr9	82,562,248	82,563,822	439	77	436	Nuclear
*CcGRAS43*	evm.model.Scaffold_78.437	62.35	4.86	45.92	85.12	−0.25	Chr8	94,136,558	94,140,430	564	195	558	Nuclear
*CcGRAS44*	evm.model.Scaffold_392.51	61.52	4.96	44.06	88.22	−0.172	Chr9	62,010,013	62,011,668	551	171	548	Cytoplasmic
*CcGRAS45*	evm.model.Scaffold_371.4	53.32	5.4	50.39	79.28	−0.301	Scaffold_371	71,514	72,770	418	47	417	Chloroplastic
*CcGRAS46*	evm.model.Scaffold_53.333	22.63	5.7	38.37	93.23	−0.076	Scaffold_53_4	86,446	87,051	201	1	199	Mitochondrial
*CcGRAS47*	evm.model.Scaffold_16.196	15.46	6.91	68.93	93.85	−0.032	Scaffold_16_2_2	187,357	187,764	135	2	127	Cytoplasmic
*CcGRAS48*	evm.model.Scaffold_60.328	62.48	4.86	46.39	84.97	−0.26	Scaffold_60_2_3	251,568	255,442	565	196	559	Nuclear

## Data Availability

The original contributions presented in this study are included in the article/Supplementary Material. Further inquiries can be directed to the corresponding authors.

## Supplementary Materials

The following supporting information can be downloaded at: https://www.mdpi.com/article/10.3390/ijms27104617/s1.

## Abbreviations

The following abbreviations are used in this manuscript:

HMM	Hidden Markov Model
MEME	Multiple Em for Motif Elicitation
qRT-PCR	Real-time quantitative reverse transcription PCR
CDS	Coding sequence
